# The Relationships between Phenolic Content, Pollen Diversity, Physicochemical Information and Radical Scavenging Activity in Honey 

**DOI:** 10.3390/molecules16010336

**Published:** 2011-01-07

**Authors:** Annamaria Giorgi, Moira Madeo, Johann Baumgartner, Giuseppe Carlo Lozzia

**Affiliations:** 1 Department of Plant Production, University of Milan, Via G. Celoria 2, 20133 Milano, Italy; 2 Department of Agri-Food and Urban System Protection and Biodiversity Enhancement, University of Milan, Via G. Celoria 2, 20133 Milano, Italy; Email: moira.madeo@unimi.it (M.M.); johann.baumgartner@unimi.it (B.J.); giuseppe.lozzia@unimi.it (L.G.C.)

**Keywords:** honey, pollen diversity, phenolic content, radical scavenging activity, multivariate analysis

## Abstract

Honey is rich in different secondary plant metabolites acting as natural antioxidants and contributing to human health. Radical scavenging activity (RSA) is related to antioxidant activity, while the correlation between the phenolic content and RSA is often weak. Consequently, exclusive information on phenolics is often insufficient to qualify the RSA and the health promoting effects of honey. The paper deals with a case study of honey samples originating from the alpine areas of Italy’s Lombardia and Veneto regions and realized by standard physicochemical and statistical analytical methods. In pure honey, the total phenolic content and the RSA were measured in spectrophotometric tests with the 2,2-diphenyl-1-picrylhydrazyl (DPPH·) free radical and Folin-Ciocalteu assays, respectively. Melissopalynological data was used to qualify pollen diversity through rank-frequency curves separating the samples into two groups. On the basis of physicochemical data, the samples were analyzed through multivariate classification and ranking procedures resulting in the identification of an outlier. Elimination of the outlier produced a high correlation between the total phenolic content and RSA in the two pollen diversity groups. The case study suggests that, after disregarding outliers, the RSA activity can be satisfactorily qualified on the basis of phenolics with pollen diversity as a covariate.

## 1. Introduction

Honey is a natural product widely appreciated as a sweetener, and for its fragrance and flavor which are due to the specific properties of the area of production and the harvesting practices [1]. Many studies have demonstrated the health-promoting effects of honey, such as reduction of heart diseases, cancer, immune system decline, and the control of different inflammatory processes [2]. The effects appear to be mainly associated with the antioxidant activities that positively affect diverse physiological processes [3,4]. As an aside, the antioxidants also play an important role in food preservation, *i.e.*, the prevention of lipid peroxidation [5]. The antioxidant activities, measurable through radical scavenging activities (RSA) [6], depend on climatic and environmental conditions as well as on the plants visited by the honey bees [7,8]. The number of plant species and their proportion visited by the bees are responsible for the diversity of pollen appearing in honey samples [7]. The RSA is associated with a great number of honey components exhibiting enzymatic and non-enzymatic activities [3,7,9]. Amongst these components are phenolics, which are of general interest in human medicine [7,10,11]. In Slovenian honeys, Bertoncelj found a high correlation between the RSA and the total phenolic content [12]. Similarly, a high correlation was detected in commercial honeys from different floral sources in Burkina Faso [13]. In contrast, Gheldof found a low correlation between RSA and phenolics [9]. The same result was reported by Meda, who analyzed artisanal honeys from different sites in Burkina Faso [3]. Baltrušaityté observed that the RSA activity in Lithuanian honey samples varied over a wide range and pointed out that the RSA depends on a great number of non-phenolic molecules including enzymatic and non-enzymatic constituents such as glucose oxidase, catalase, ascorbic acid, organic acids, Maillard reaction products, amino acids and proteins [7]. The diversity of pollen may be very different in these samples and explain the inconsistently of the results [3]. 

Taken together these studies indicate that it is difficult to draw general conclusions on health promoting effects in general and antioxidant activity in particular, if the available information is restricted to phenolics. In a case study, the variability of honey samples from the alpine areas of Italy’s Lombardia and Veneto regions was studied. The samples are treated as experimental data for developing and evaluating methods that permit the qualification of antioxidant activity and RSA of honey samples on the basis of pollen diversity, phenolic content, melissopalynological information and physicochemical measurements.

## 2. Results and Discussion

### 2.1. Variability assessment through melissopalynological analyses

Table 1 lists the percentage of pollen from different plant species in the samples and shows that 99% of the total pollen was supplied by 4-10 plants. The remaining 1% of the total pollen originated from various other plants and is disregarded in this work. Among the 11 samples, 10 were classified as multifloral honey consisting of a mixture of pollen from different plant species, while sample L was a monofloral chestnut honey. Two samples were produced in the Veneto region, while the other samples originated in the Lombardia region (Table 1). The melissopalynological analysis of the L and M samples confirmed the classification given on the label. 

**Table 1:** List of samples, sample codes, region of origin, locations, data of collection, and percentages of the most important pollen supplying plants.

Sample code	Origin	Location	Data of collection	Pollen supplying plants (%)
A05	Lombardia region	Monno (1066 m a.s.l.)	June 2005	*Castanea* (78.2), *Ericaceae* (12.7), *Rubus* (4.4), *Achillea* (1.8), *Pyrus* (1.7), *Trifolium* (0.4)
B05	Lombardia region	Monno (1066 m a.s.l.)	July 2005	*Castanea* (75.2),*Ericaceae* (10.7), *Rubus* (3.9), *Achillea* (0.7), *Tilia* (0.4), *Salix* (0.3)
C06	Lombardia region	Monno (1066 m a.s.l.)	August 2006	*Rhododendron* (42), *Rubus* (30), *Cruciferae* (5.4), *Trifolium* (4.6), *Salix* (2.5),*Robinia* (1.8), *Tilia* (1.4), *Umbelliferae* (0.7)
D05	Lombardia region	Saviore dell’Adamello (1210 m a.s.l.)	July2005	*Castanea* (65.3), *Rhododendron* (16.5), *Rubus* (12.7), *Trifolium* (1.3), *Salix* (1.0), *Myosotis* (0.6), *Knautia* (0.4)
E06	Lombardia region	Saviore dell’Adamello (1210 m a.s.l.)	August 2006	*Rubus* (46.1), *Rhododendron* (38,4), *Trifolium* (3.1), *Myosotis* (2.9), *Compositae* (1.4), *Achillea* (1.9), *Silene* (1.0)
F05	Lombardia region	Vezza d’Oglio(1080 m a.s.l.)	July 2005	*Rhododendron* (80.0), *Cruciferae* (7.4), *Rubus* (2.9), *Trifolium* (1.3), *Castanea* (1.3), *Umbelliferae* (1.1), *Astragalus* (0.8)
G06	Lombardia region	Vezza d’Oglio (1080 m a.s.l.)	May 2006	*Myosotis* (70), *Taraxacum* (31.8), *Prunus* (21.7), *Lotus* (14.3), *Salix* (10.4), *Acer* (6.4), *Rubus* (3.3), *Sorbus* (3.3)
H06	Lombardia region	Vezza d’Oglio (1080 m a.s.l.)	June 2006	*Trifolium* (30.0), *Robinia* (18.2), *Rhododendron* (14.0), *Rubus* (13.6), *Myosotis* (7.9), *Sorbus* (5), *Prunus* (1.1)
I06	Lombardia region	Vezza d’Oglio (1080m a.s.l.)	July 2006	*Trifolium* (30.0), *Myosotis* (28.9), *Umbelliferae* (11.8), *Rubus* (5.1), *Salix* (4.2), *Acer* (3.2), *Salvia* (2.4), *Lotus* (2.4)
L	Veneto region	not available	2006	*Castanea* (92), *Rubus* (2,4), *Trifolium* (2,2), *Eucalyptus* (2.0)
M	Veneto region	not available	2006	*Salix* (28.5), *Acer* (27), *Prunus* (13.7), *Sorbus* (9.8), *Robinia* (4.6), *Aesculus* (4.3), *Trifolium* (3.1), *Amorpha* (3.0), *Rubus* (1.5)

* Sample L, marked in grey was qualified as a monofloral honey, while all the other samples are qualified as multifloral honeys.

The samples A05, B05, D05 contained a high percentage of the most representative pollen types (70-80%). *Castanea,* *Ericaceae,* *Rubus* were the dominant pollen types in honeys A05 and B05. Sample L, however, contained 92% of *Castanea* pollen. Pollen from *Rhododendron, Rubus* and *Trifolium* was present at significant levels in the C06, D05, E06, F05 and H06 samples. *Myosotis, Taraxacum,* and *Prunus* were the main contributors of the pollen to sample G06. *Myosotis* pollen was as also present in D05, E06, H06 and I06. The highest number of plants supplied the pollen in sample M, with *Salix, Acer,* and *Prunus* being the important ones. *Rubus* pollen was present in all samples but differed in its percentages. Similarly, *Trifolium* pollen occurred in all samples with the exception of the sample G06. Our results indicated that the distribution of the pollens greatly vary among honey samples confirming the influence of the environmental conditions [1,14]. 

**Figure 1:**
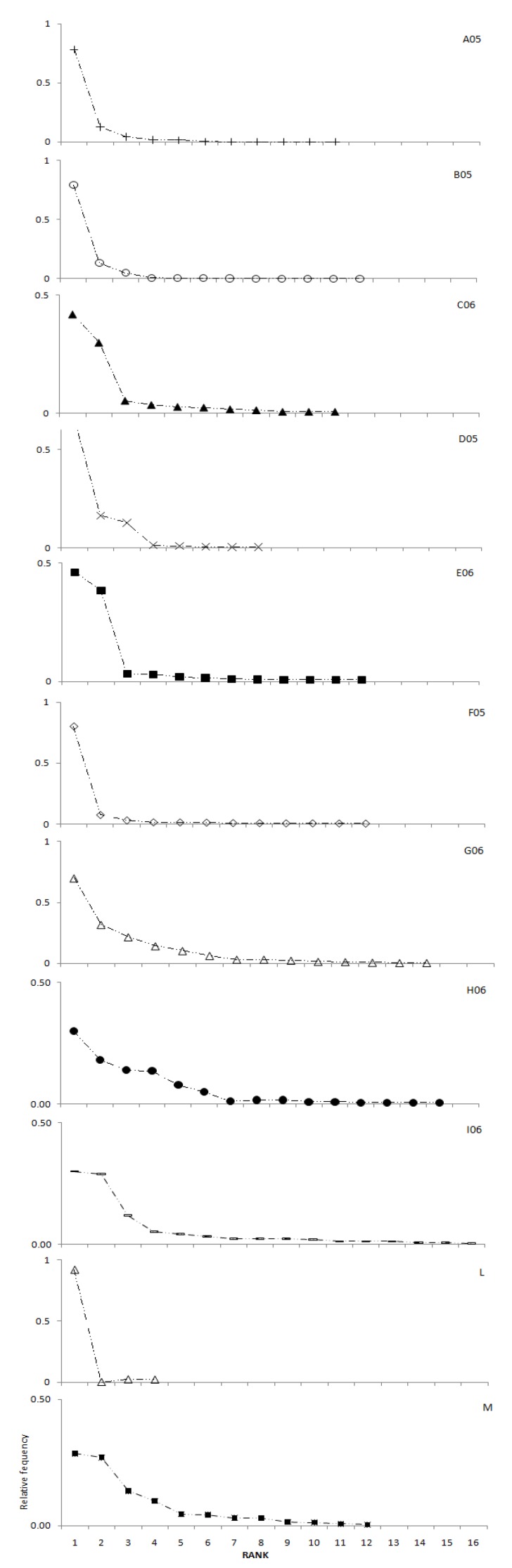
Pollen diversity represented by the relative frequency and the rank in the honey samples (A05, B05, C06, D05, E06, F05, G06, H06, I06, L, M). Unfilled symbols represent the low diversity group 1 that is dominated by pollen from a restricted number of few plant species. Filled symbols represent the high diversity group 2 samples with pollen from many plants and an even representation of plants.

Figure 1 depicts the diversity of pollen represented by rank-relative frequency diagrams. The ordinate represents the ranking of the 11 samples, while the abscissa represents the relative frequency of pollen of different plants in the samples. The samples appear to fall into two groups. Group 1 consists of the samples A05, B05, D05, F05, G06 and L that are dominated by pollen from a few plant species (Table 1). It is noteworthy that the monofloral sample L, containing 92% of *Castanea* pollen, appears in this group. Group 2 consists of the remaining samples C06, E06, H06, I06 and M, where more plants contributed to the pollen. The multifloral samples H06 and I06 contain the pollen distribution with the highest evenness.

### 2.2. Variability assessment through physicochemical measurements, total phenolic content and radical scavenging activity

Table 2 lists the physicochemical measurements, the total phenolic content and the RSA of the honey samples. The physicochemical measurements of all samples were within the limits stipulated by the European Honey Directive (15). The total phenolic content was similar to the one reported by Meda [3]. The RSA values are also in a similar range, as previously reported [3,12].

**Figure 2:**
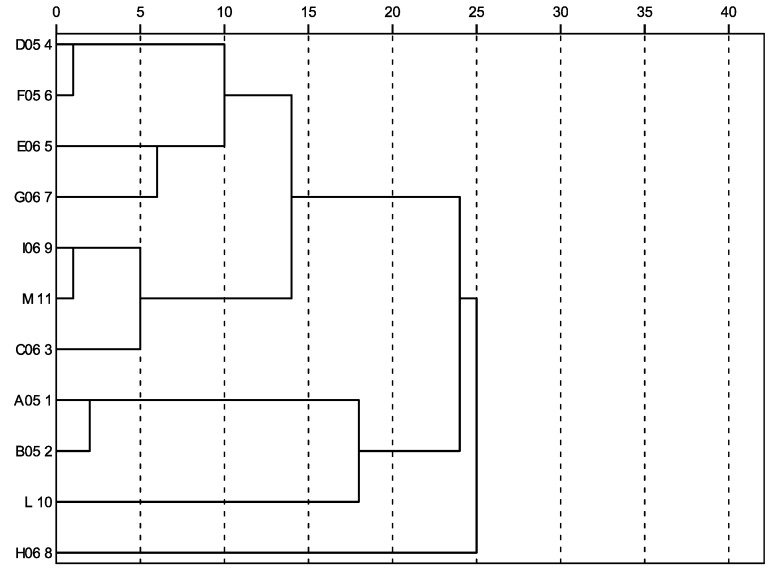
Dendrogram of the honey samples (ordinate), based on physicochemical measurements, total phenolic contents and radical scavenging activity (RSA). The similarity level is represented on the abscissa.

Figure 2 shows the grouping of the samples according to the hierarchical cluster analysis. A first group delimited at the similarity level of 20% included the samples A05, B05 and L with the highest phenolic contents (values reported in Table 2). Notably, the samples A05 and B05 grouped at the 1% level were harvested at the same locality in June and July 2005. These two samples were classified as multifloral honeys characterized by a high content of *Castanea* pollen, which could explain the similarity to the chestnut honey L (values reported in Table 2). A second group included the commercial multifloral sample M and the artisanal samples I06 and C06 collected in the same year but at different locations. These samples fall into the same range of total phenolic content and the RSA (Table 2). The samples D05, E06, F05 and G06 are members of the third group, grouped at the 2% similarity level. Importantly, the cluster analysis excludes the H06 sample from the other groups, while the aforementioned rank frequency analysis assigned it to the second group. This sample shows the lowest total phenolic content, the highest RSA and the lowest conductivity (values reported in Table 2).

**Table 2:** Physicochemical measurements, total phenolic content, radical scavenging activity (RSA) (IC_50_) of honey samples.

Sample	pH	Free acidity (meq/kg)	Conductivity (10^-4^ S cm^-1^)	Moisture (%)	Total phenolic content (mg GAE/100g ± SD)	Radical Scavenging Activity (RSA) IC_50_ (mg/mL ± SD)
A05	4.66	26	6.77	16.5	62.80 ± 2.09	16.14 ± 0.73
B05	4.60	26	7.38	18.5	57.79 ± 4.89	12.03 ± 0.35
C06	4.04	29	4.70	17.3	25.31 ± 0.61	63.23 ± 1.24
D05	4.05	14	2.30	17.3	43.12 ± 3.32	52.28 ± 0.31
E06	4.51	23	6.10	16.3	34.13 ± 2.98	21.30 ± 1.39
F05	3.85	9	2.59	16.8	36.85 ± 4.74	44.06 ± 0.08
G06	4.41	18	5.40	18.1	32.22 ± 4.53	20.62 ± 1.87
H06	3.85	13	1.50	16.4	15.13 ± 3.93	11.39 ± 2.11
I06	3.88	23	3.30	16.9	29.06 ± 7.32	56.36 ± 3.90
L	5.21	21	16.80	17.0	82.49 ± 4.05	19.00 ± 1.84
M	4.34	23	7.84	16.7	24.21 ± 1.43	64.33 ± 2.76

**Figure 3:**
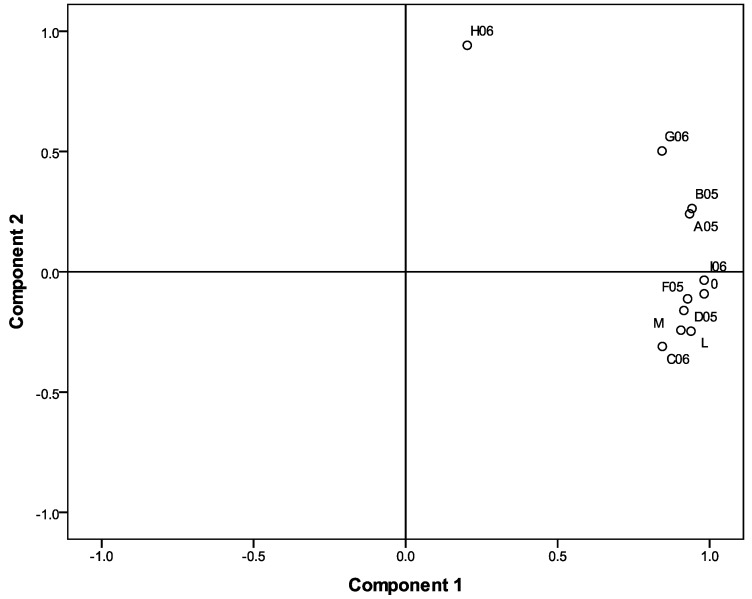
The honey samples separated by Principal Component Analysis (PCA) on the basis of physicochemical variables, phenolic content and radical scavenging activity (RSA). (The E06 sample is represented by the dot close to the I06).

Figure 3 shows the grouping of the samples according to the Principal Component Analysis. Bartlett’s sphericity test resulted in a statistical significance (p < 0.001), indicating the suitability of the matrix for the PCA [16]. First, the PCA was performed considering the samples with respect to their components. The procedure extracted two principal components explaining 77.4% and 15.1% of the total variance for PC1 and PC2, respectively (Figure 3). In PC1, all variables showed a positive value, indicating a positive correlation. In PC2, the samples C06, D05, E06, F05, I06 and L showed a negative value meaning a negative correlation (Figure 3). In particular, the sample H06 appearing on the top of the right-hand side of Figure 3 is separated from the other samples. This may be due to relatively low values for the physicochemical variables, phenolic content and radical scavenging activity (RSA) (Table 2). The same variables are responsible for the separation of the samples on the PC2 axis. 

Both the cluster and principal component analysis separated the H06 sample from the other samples. For this reason, this sample is considered an outlier. Currently, there is no information available that could be used to explain the qualities of this sample. According to the grouping and the separation of the other samples, the results of the PCA generally correspond to the CA. The most conspicuous exceptions are the samples L and G06 which are associated with different samples in the two multivariate analyses. We performed both PCA and CA to exploit the complementarity of the methods for analyzing the structure of the samples. The two methods rely on either ordination or classification of data and hence, produce slightly different results that do not affect the subsequent correlation analysis.

### 2.3. The relationship between radical scavenging activity (RSA) and total phenolic content

We correlated the RSA expressed as 1/IC_50_ with the total phenolic content. The correlation coefficients (R^2^) between RSA and total phenolic content found for pure honey was equal to 0.679. This low correlation coefficient was in agreement with the results obtained by other authors [3,7,9]. Importantly, the elimination of the outlier H06 resulted in R^2^=0.878. Based on the grouping of the rank-relative frequency analysis, we performed a correlation analysis for each of the two groups. In group 1 (A05, B05, D05, F05, G06, L) we found a high correlation (R^2^=0.906). The Pearson’s correlation coefficient was slightly lower in group 2 (C06, E06, H06, I06, M) (R^2^=0.870), but increased considerably when the outlier was disregarded (R^2^= 0.990). 

In summary, pollen diversity, *i.e.* the number of pollen supplying plants and the number of pollen per plant, separates the samples into the two groups, while clustering and PCA permitted the identification of an outlier. The results show that: i) the grouping is due to pollen diversity, ii) in the groups, phenolic content and RSA are highly correlated, and iii) the elimination of an outlier produced a higher correlation between RSA and phenolic contents in the specific group. However, the biochemical basis underlying the correlation between RSA and phenolics, with pollen diversity as a covariate, is unclear. The results have been obtained from an analysis of pure honey. Nevertheless, the analysis of water and acetate extracts produced similar results (M. Madeo, unpublished data) suggesting that the analysis of honey can be based on pure samples without any loss of information. The health promoting effects resulting from RSA and the related antioxidant activity cannot be directly derived from exclusive knowledge on phenolics. Adequate knowledge on pollen diversity from melissopalynological analyses is indispensable for qualifying the honey samples. Multivariate clustering and ordination procedures relying on physicochemical information are useful for detecting outliers. The elimination of outliers and the use of pollen diversity as a covariate may open the door for a qualification of honey samples with respect to health promoting effects. Since physicochemical information is needed for outlier identification, measurements on phenolics and pollen diversity are insufficient and should be completed with physicochemical information for honey qualification. 

## 3. Experimental

### 3.1. Honey samples

The study was carried out using 11 honey samples. In 2005 and 2006, nine samples (from A to I, Table 1) were purchased from local small scale beekeepers working above 1,000 m a.s.l. in the Valcamonica valley of the Lombardia region. Samples L and M (Table 1), labelled as chestnut and multifloral honey, originated in the Veneto region and were purchased in a supermarket. All samples were stored in darkness at temperature of 4-6 °C prior to analysis. 

### 3.2. Melissopalynological analysis and physicochemical variables

Melissopalynological analysis was performed according to the techniques of the International Commission of Bee Botany (ICBB) and published in 1978 [17]. The microscopic analysis of honey sediment composition provides the percentage of the specific pollen observed by microscopic comparison with known pollen grains (Table 1). Physicochemical variables were measured according to the European Honey Commission methods and provided information on moisture, pH, acidity and electrical conductivity [18,19]. 

### 3.3. Assessment of total phenolic content

The phenolic contents of the pure honey were measured according to the method proposed by Singleton [20], that employs the Folin-Ciocalteu reagent, with gallic acid (10-200 µg/mL), to produce standard curves [3]. Three replicates were obtained from each samples for chemical analysis. Solutions of honey (100 mg/mL) were made up in distilled water, and an aliquot of each solution (0.1 mL) was mixed with 0.2 N Folin-Ciocalteau reagent (0.5 mL) and sodium carbonate (Na2CO3, 0.4 mL, 75 g/L) was added within 8 minutes. A blank reagent using distilled water was also prepared. After incubation at 40°C for 30 minutes the absorbency could be followed in a spectrophotometer at 765 nm (UV/VIS spectrophotometer, Jasco-7800; Milan, Italy). Phenolic compounds were calculated by absorbance values obtained from standard curves constructed by plotting the absorbance against concentration of standard gallic acid solutions, and therefore expressed as gallic acid equivalent (GAE).

### 3.4. Radical scavenging activity (RSA)

The RSA against 2,2-diphenyl-1-picrylhydrazyl (DPPH·) radical was assessed according to the previously described method [3] with minor modifications. Three replicates were obtained from each samples for chemical analysis. In the presence of a radical scavenger, the purple colour of (DPPH·) radical decays, and the change of absorbance was measured at 517 nm, using an UV/VIS spectrophotometer (Jasco-7800; Milan, Italy) and ascorbic acid as standard. Pure honey (100 mg/mL) was dissolved in methanol and an aliquot of each solution (0.1 mL) was mixed with DPPH· in methanol (0.8 mL, 0.02 mg/mL), using methanol as a blank sample. The mixture was left for 15 minutes in the dark at room temperature. Radical scavenging activity was calculated as difference between the absorbency of (DPPH·) methanol solution without sample (A_A_) and (DPPH·) in contact with different concentrations of sample (A_B_). The inhibition percentage of the radical (I_%_) was calculated by the formula:

I_%_ = [(A_A_-A_B_)/A_B_] x 100

The concentrations versus the inhibition percentages, IC_50_ values (concentration causing 50% inhibition) were calculated. Smallest values of IC_50 _correspond to higher radical scavenging activity. 

### 3.5. Statistical analysis

Two statistical procedures are applied to qualify the honey samples reported in Table 1. First, the melissopalynological data were used to analyze the diversity of pollen represented by rank-relative frequency curves widely used in ecological work [e.g., 21,22]. Briefly, this methods allows the representation of the number of pollen supplying plants (through ranking) and of the contribution of individual plants to the pollen samples (through the relative frequency). To generate the relative frequency curves for each sample, we ordered all pollen species (from 1 to 21), calculated the relative pollen frequency for each species, *i.e.*, the proportional number in the entire samples, and plotted the relative frequency against the rank. The shape of the curves provides information on the degree of evenness of pollen distribution of the samples. 

Second, the samples were further analyzed through multivariate clustering and ordination procedures on the basis of physicochemical measurements, total phenolic content and RSA. Here, the hierarchical cluster analysis (CA) uses the Euclidean distance as a measure of similarity and permits the grouping of data in a dendrogram [16], while the Principal Component Analysis (PCA) reduces the variables to the first two components explaining most of the variability. The multivariate methodology and its application to ecological studies has been detailed, for example, by Gauch [23]. 

Third, we calculated the Pearson's correlation coefficients between RSA and phenolic contents for the major groups obtained in the pollen diversity study. This procedure was repeated after eliminating outliers identified in CA and PCA analyses. Statistical analysis was carried out using SPSS for Windows version 18.0 statistical package .

## 4. Conclusions

The combination of standard physicochemical and statistical methods allows the analyses of heterogeneous honey samples. Melissopalynological information is useful to study pollen diversity represented in rank-frequency curves. The shape of these curves allows a grouping of samples. On the basis of phenolic contents, RSA and physicochemical data, CA and PCA can be employed to detect possible outliers. After the elimination of outliers, satisfactory correlation coefficients between radical scavenging activity and phenolic contents can be found for each pollen diversity group. After disregarding outliers, the RSA and antioxidant activities can be assessed if pollen diversity is taken into account as a covariate. 

To better understand the health promoting quality of honey, we recommend obtaining melissopalynological information, physicochemical measurements and data on phenolic contents and subjecting these data to pollen diversity and multivariate statistical analysis. After the elimination of outliers, the qualification of honeys could be based on phenolics with pollen diversity as a covariate. These results have been obtained for samples of a specific region. A comparison with the variability of honey samples reported in the literature; however, suggest a possibility for extending the results to other areas. 
